# Intra-specific variations in *Schistosoma mansoni* and their possible contribution to inconsistent virulence and diverse clinical outcomes

**DOI:** 10.1371/journal.pntd.0012615

**Published:** 2024-10-28

**Authors:** Tim A. Dannenhaus, Franziska Winkelmann, Cindy Reinholdt, Miriam Bischofsberger, Jan Dvořák, Christoph G. Grevelding, Micha Löbermann, Emil C. Reisinger, Martina Sombetzki

**Affiliations:** 1 Division of Tropical Medicine and Infectious Diseases, Center of Internal Medicine II, Rostock University Medical Center, Germany; 2 Institute of Organic Chemistry and Biochemistry, Czech Academy of Sciences, Prague, Czechia; 3 Department of Ecology, Center of Infectious Animal Diseases, Faculty of Environmental Sciences, Czech University of Life Sciences, Czechia Institute of Parasitology, Prague, Czechia; 4 Biomedizinisches Forschungszentrum Seltersberg, Justus Liebig University Giessen, Giessen, Germany; James Cook University, AUSTRALIA

## Abstract

**Background:**

*Schistosoma mansoni* was introduced from Africa to the Americas during the transatlantic slave trade and remains a major public health problem in parts of South America and the Caribbean. This study presents a comprehensive comparative analysis of three *S*. *mansoni* strains with different geographical origins—from Liberia, Belo Horizonte and Puerto Rico. We demonstrated significant variation in virulence and host-parasite interactions.

**Methods:**

We investigated the phenotypic characteristics of the parasite and its eggs, as well as the immunopathologic effects on laboratory mouse organ systems.

**Results:**

Our results show significant differences in worm morphology, worm burden, egg size, and pathologic organ changes between these strains. The Puerto Rican strain showed the highest virulence, as evidenced by marked liver and spleen changes and advanced liver fibrosis indicated by increased collagen content. In contrast, the strains from Liberia and Belo Horizonte had a less pathogenic profile with less liver fibrosis. We found further variations in granuloma formation, cytokine expression and T-cell dynamics, indicating different immune responses.

**Conclusion:**

Our study emphasizes the importance of considering intra-specific variations of *S*. *mansoni* for the development of targeted therapies and public health strategies. The different virulence patterns, host immune responses and organ pathologies observed in these strains provide important insights for future research and could inform region-specific interventions for schistosomiasis control.

## Author summary

Schistosomiasis continues to be a major global health problem, affecting millions of people worldwide. One of the human pathogens, *Schistosoma mansoni*, was introduced to the Americas from Africa during the transatlantic slave trade. Despite its widespread distribution, intraspecific variation between geographically separated strains of *S*. *mansoni* has been studied insufficiently. Our study provides a comparative analysis of three laboratory *S*. *mansoni* strains with original provenance from Liberia, Belo Horizonte and Puerto Rico. We found significant intraspecific differences in virulence patterns, host immune responses and organ pathology. Our results show differences in the phenotypic characteristics of the parasite and its eggs. The Puerto Rican strain was the most virulent and was characterized by severe liver and spleen damage and advanced liver fibrosis. In contrast, the strains from Liberia and Belo Horizonte were less pathogenic and showed only minimal liver fibrosis. In addition, we observed significant differences in granuloma formation, cytokine expression and T-cell dynamics, indicating different host immune responses. These different virulence patterns and immune responses highlight the need to consider the intraspecific variation of *S*. *mansoni* when assessing disease burden and developing new therapeutic approaches.

## Introduction

Schistosomiasis, caused by digenetic trematodes of the genus *Schistosoma* spp., affects over 250 million individuals in tropical regions globally [1]. As one of the neglected tropical diseases, schistosomiasis poses a major public health problem and approximately 1.9 million disability-adjusted life years could be attributable to the infection [2]. All schistosome species share a complex life cycle, with obligate alternation between vertebrate and non-vertebrate hosts. Each *Schistosoma* species has a strong specificity for its hosts. In the case of *Schistosoma* (*S*.) *mansoni*, humans, wild rodents and non-human primates (mice and hamsters in experimental settings) represent the definitive hosts (sexual maturation of the adult worms) and the freshwater snail from the genus *Biomphalaria* the intermediate host (asexual reproduction). Schistosomes are transmitted via skin contact with water contaminated by free-swimming *Schistosoma* larvae (cercariae), which are shed by freshwater snails. After skin penetration, larvae convert into schistosomula, migrating via the lymphatic system through the heart and lungs to the liver’s vasculature for mating and maturation [3]. Post-mating, they move against the blood flow to the mesenteric blood vessels to start laying eggs. Schistosomes have the ability to survive within their definitive hosts, including humans, for an average of 3–10 years [4,5]. Adult female *S*. *mansoni* can produce approximately 300 eggs daily [6], which are deposited within the mesenteric vasculature, adhering to the endothelial lining of capillary walls [7]. About half to two-thirds of these eggs are disseminated through the bloodstream to other organs, primarily the liver [8]. The metabolically active and highly antigenic eggs induce granulomatous inflammation and subsequent fibrosis. These factors contribute to significant pathologies associated with *S*. *mansoni*, including obstructive portal lesions, portal hypertension, hepatic encephalopathy, variceal hemorrhage, and ascites [9,10]. Conversely, eggs that migrate through the intestinal epithelia into the intestinal lumen also incite granuloma formation. In the intestine, this reaction is essential for their translocation through the lamina propria, leading to their eventual excretion in feces, thereby completing the mammalian host component of the life cycle [6].

The risk of infection is highest for school-age children due to a lack of immunity, poor sanitation and their activities near or in fresh water [5]. As global warming creates suitable conditions for the survival of snail intermediate hosts in previously or longtime uncolonized areas, the spread of schistosomiasis could increase considerably in the future [11], as shown by the more than 100 cases of urogenital schistosomiasis in Corsica in 2014 (France) [12]. Praziquantel, the only therapeutic agent available for this disease [13], primarily targets the adult stages of the parasite and does not prevent reinfections [14]. The efficacy of mass drug administration (MDA) varies with the endemicity of the region [15]. Relying heavily on MDA using only praziquantel may raise concerns about potential drug resistance in schistosomes [16–19].

*S*. *mansoni* was predominantly found in Africa and the Middle East and is thought to have been introduced from Africa to the New World during the transatlantic slave trade between the 16th and 19th centuries. The forced migration of innumerable people facilitated the spread of *S*. *mansoni* in America. The synergistic effects of ecological conditions, such as the presence of suitable snail hosts from the genus *Biomphalaria*, and the insanitary conditions during the slave trade, created an optimal environment for the transmission and establishment of the disease [20–22]. Interestingly, molecular evidence suggests a distinct historical trajectory for the intermediate host *Biomphalaria glabrata*. It posits that a *B*. *glabrata*-like taxon likely originated in South America. Subsequently, it is believed to have migrated to Africa during the Plio-Pleistocene epoch (1.8–3.6 million years ago) via waterfowl feathers [23]. The introduction of *S*. *mansoni* in America had a significant public health impact and resulted in schistosomiasis becoming a major health burden in parts of South America and the Caribbean [24]. *S*. *mansoni* is the causative agent for the disease in more than 54 million individuals, affecting regions like sub-Saharan Africa, the Caribbean islands, Puerto Rico, Suriname, Venezuela, and Brazil [25].

The geographic origin of *S*. *mansoni* strains significantly influences their biological characteristics, thereby affecting the variability in their interactions with the host [26]. This variability manifests in different ways. For example, strains from different regions show differences in infectivity, which is reflected by variations in penetration rate, adult worm presence, and the duration of the prepatent period [27–30]. There are also notable differences in egg production, distribution, and excretion patterns [27–32]. Geographic and sub-strain variations play an important role in determining pathobiological characteristics such as granuloma formation, fibrosis, and survival time [28,30,31,33]. In particular, strains from the Americas show increased virulence and more pronounced disease symptoms than those from Africa [32]. The interaction between parasite genetics, which includes aspects like oviposition and intestinal wall migration, and host genetics plays a key role in shaping infection outcomes [29].

Given the observed genetic variations in present-day *S*. *mansoni* strains across different regions potentially attributed to historical transmigration, there are consequent variations in virulence and clinical outcomes [34,35]. These disparities underscore the necessity for comprehensive phenotypic evaluations of *S*. *mansoni* strains. Such assessments enable precise characterization of the strains and also provide important information for predicting infection outcomes, making it possible to formulate region-specific control strategies [36]. To fully address these questions, a profound exploration of intraspecific variations within *S*. *mansoni* is needed, since intra-specific variations might bear significant implications for the parasite’s virulence and the effectiveness of therapeutic interventions [37]. A comprehensive understanding of the different genetic and phenotypic traits that influence virulence can contribute to the refinement of diagnostic tools, improve control measures and promote the development of innovative therapeutic and preventive strategies [36].

In this comparative study, we examine three geographically distinct *S*. *mansoni* strains, intending to enhance our comprehension of the host-parasite interaction. We postulate that these strains have unique phenotypic characteristics that influence their virulence. Furthermore, we hypothesize that these distinctions induce different and immunopathological responses in the host that influence the course of the infection. The three *S*. *mansoni* strains chosen for our study originate from Liberia, Belo Horizonte (Brazil), and Puerto Rico. Based on the available literature, we assume that the strains from Belo Horizonte and Puerto Rico have a higher virulence than the Liberian strain. However, we would like to point out directly that all three strains are parasites that have been kept in laboratories for a prolonged period of time and not new isolates from the wild.

## Materials & Methods

### Ethics statement

Animal experiments were conducted in strict compliance with the regulations of the German Society for Laboratory Animal Science and with the European health guidelines issued by the Federation of Laboratory Animal Science Associations. The protocol was approved by the local animal research committee (Mecklenburg-Western Pomerania State Office for Agriculture, Food Safety and Fisheries, ref. number M-V/TSD/7221.3-2-007/18-3). All efforts were made to minimize animal suffering. Ketamine/xylazine anesthesia followed by cervical dislocation were employed to sacrifice the mice as indicated below.

### *Schistosoma mansoni* infection mouse model

**Fig 1:**
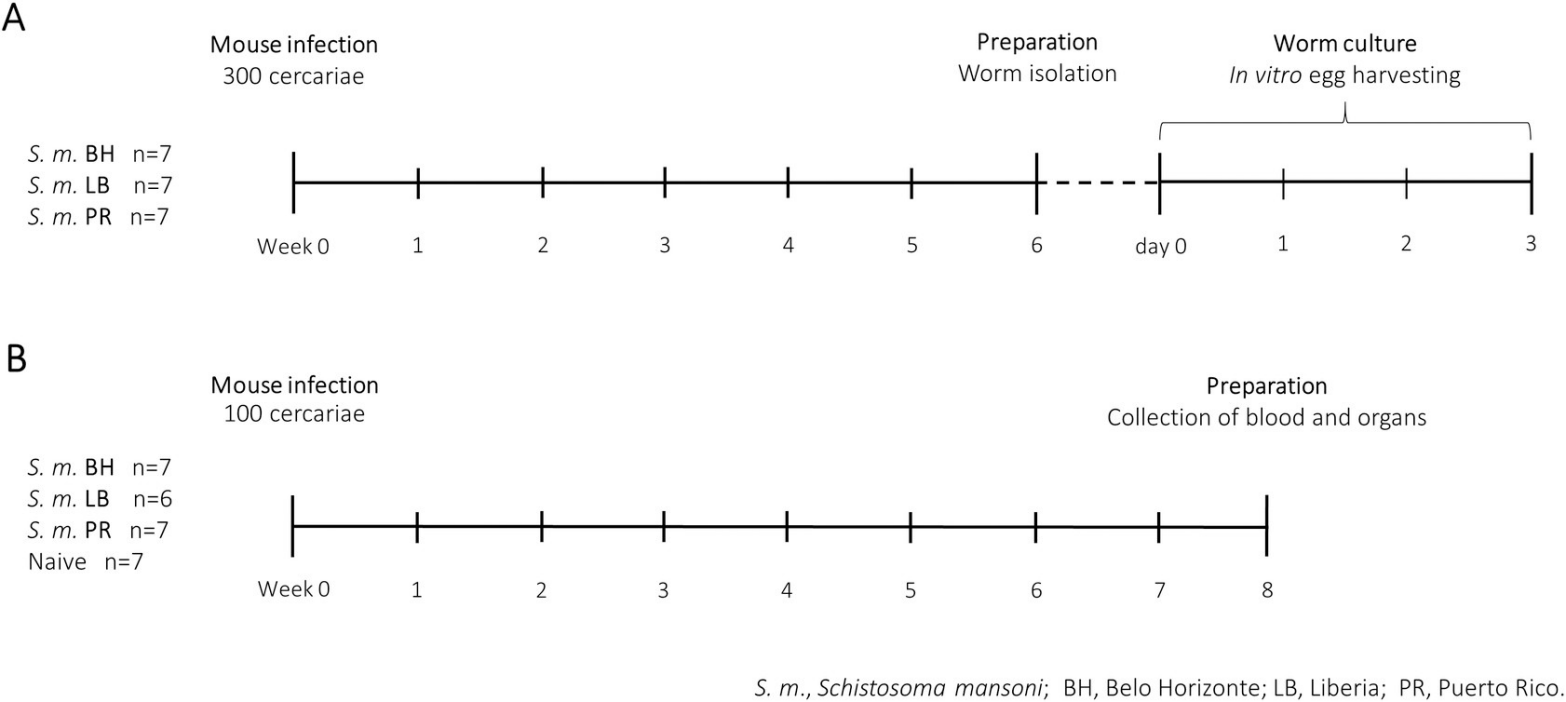
Experimental timeline. We investigated the effects of different *Schistosoma mansoni* strains on host pathology. (A) Groups of seven NMRI mice were each infected with 300 cercariae of the BH, LB or PR strain. 42 days after infection, the mice were perfused to extract adult worms from the portal vein system and the body size of the worms was measured. Subsequently, the extracted worms were cultured in vitro to evaluate their egg-laying ability. (B) We used another set of seven mice per strain and infected them with 100 cercariae each (one mouse infected with the LB strain was prematurely excluded from the study). These mice were euthanized 56 days after infection, and we examined the pathological effects on the liver and spleen as well as the changes in serum blood levels. Belo Horizonte, BH; Liberia, LB; Puerto Rico, PR.

Laboratory *S*. *mansoni* strains (Belo Horizonte, BH; Liberia, LB; Puerto Rico, PR) were kept in life cycles using *B*. *glabrata* fresh water snails as intermediate hosts and 6–8 weeks old female NMRI mice as definitive hosts, as previously described [38]. The study included 49 mice in total, of which seven served as uninfected naive controls. 21 mice, with seven per *S*. *mansoni* strain (BH, LB, PR), were percutaneously infected with 300 cercariae with an assumed equal mix of male and female cercariae to analyze the parasite phenotype. On day 42 post infection (dpi), mice were sacrificed via cervical dislocation under ketamine/xylazine anesthesia. Worms were collected by perfusion of the portal venous system, as previously described (Fig 1A) [39]. After perfusion, the livers were taken for the isolation of mature eggs as described below. 56 dpi, mice (infected with 100 *S*. *mansoni* cercariae or uninfected control) were sacrificed via cervical dislocation under ketamine/xylazine anesthesia. All mice were subsequently weighed using a precision scale (Kern & Sohn GmbH, Balingen-Frommern, Germany). Blood, livers, spleens and intestines were taken, organs were imaged and weighed, related to body weight of mice, and collected for further analysis as described below (Fig 1B). One mouse infected with the LB strain was sacrificed before the end of the study due to poor general health and therefore excluded from the study.

### Analysis of the parasite phenotype

Collected worms from the portal vein were washed three times with washing buffer (RPMI with 100 U/ml penicillin and 100 μg/ml streptomycin) and counted under the stereomicroscope (males, females, juveniles). Intact adult worms were either fixed for morphological analyses or used for *in vitro* cultivation. For morphological analyses only mated worms were used, which were subsequently separated by placing them in a 4°C environment for 20 min. Twentyone worms of each sex and strain were fixed in 4% formaldehyde solution and body length was analyzed by ImageJ (v. 1.53, National Institutes of Health, USA). To assess the egg laying (oviposition) capacity, already paired worms were placed into 24-well plates (1 pair/well), and cultivated as described before [40]. Egg production was counted after 3-day cultivation at 37°C in 5% CO_2_. Further, length of *in vitro* laid immature eggs was analyzed by ImageJ (v. 1.53). Since immature eggs isolated from the host tissue are difficult to distinguish from dead eggs, eggs laid *in vitro* were used to ensure the immaturity of the eggs. To analyze the length of mature eggs, they were isolated from livers as described before [41] and length was measured by ImageJ (v. 1.53).

### Egg isolation from liver

Standardized liver lobes were incubated in 4% potassium hydroxide (KOH; 37°C, shaking 500 rpm) overnight. After centrifugation (5 min, 100 x g), the pellets were resuspended in 400 μl of PBS and eggs were counted in 3 x 20 μl aliquots examined under the microscope.

### Liver histology and hepatic hydroxyproline content

Liver samples from mice, infected for 56 days, were fixed in 4% neutral buffered formalin solution. These were subsequently embedded in paraffin and sectioned to a thickness of 4 μm. Sections were stained with either hematoxylin/eosin (H&E) or sirius red (SR). Ten granulomas per mouse were analyzed. Granuloma dimensions in the H&E-stained sections were quantified using ImageJ software (v1.47v; National Institutes of Health, USA). The total amount of collagen in a defined liver section was quantified based on colorimetric detection of hydroxyproline using a Quickzyme Total Collagen Assay Kit (Quickzyme Biosciences, Netherlands) according to the manufacturer’s instructions.

### Serum biochemistry

Serum biochemical analyses of alanine aminotransferase (ALT), aspartate aminotransferase (AST), and alkaline phosphatase (AP) were performed using the UniCel DxC 800 Synchron Clinical System (Beckman Coulter GmbH, Germany).

### Quantitative real-time-PCR (qRT-PCR) analysis of gene expression

Total RNA was isolated (RNeasy Plus Mini Kit, Qiagen, Hilden, Germany) from snap-frozen standardized liver lobes and reversely transcribed into cDNA (High-capacity cDNA Reverse Transcriptase Kit, Thermo Fisher Scientific, Erlangen, Germany) according to the manufacturer’s instructions. qRT-PCR was performed using following TaqMan Gene Expression Assays: *acta-2* (Mm00725412), *col1ɑ2* (Mm00483888_m1), *ifn-γ* (Mm01168134), *il-1β* (Mm00434228), *tnf-α* (Mm00443258), *il-12a* (Mm00434169), *il-5* (Mm00439646_m1), and *il-13* (Mm00434204) (Thermo Fisher Scientific, Erlangen, Germany). The analyses were performed by QuantStudio 3 with the following reaction setup: 50°C for 2 min followed by 95°C for 10 min, 45 cycles at 95°C for 15 sec, and at 60°C for 1 min. Gene expression data were normalized to endogenous control *gapdh* (Rodent GAPDH Control Reagents, Thermo Fisher Scientific, Erlangen, Germany) and are shown in relation to the naive mice.

### Flow cytometry

Single cell suspensions were prepared from whole spleens and a defined fraction of the liver. The livers had to be digested beforehand. This was done by mincing the tissue and incubating it in 4 ml of RPMI Medium Complete (RPMI 1640 containing 10% FCS, 25 mM HEPES and penicillin/streptomycin (penicillin: 100 U/ml, streptomycin: 100 μg/ml) supplemented with 1 mg/ml collagenase/dispase (Sigma Aldrich, Darmstadt, Germany) (collagenase: 0.1 U/ml, dispase: 0.8 U/ml) for 30 min at 37°C. The predigested livers and the spleens were then passed through a cell strainer (100 μm and 70 μm) and rinsed with PBS, followed by lysis of the erythrocytes with red blood cell lysis buffer (BioLegend, London, UK). Cells were washed with PBS and cell number was quantified using a CASY TT cell counter (OLS-Omni Life Science, Bremen, Germany).

A total of 10^6^ cells/sample were stained with a Zombie NIR Fixable Viability Kit (BioLegend, London, UK) for 20 min at RT in PBS followed by a 20-min incubation with mouse anti-CD16/32 (clone: 93; BioLegend, London, UK) and stained with appropriate fluorochrome-conjugated surface marker antibodies: VioGreen anti-CD45 (clone: REA747; Miltenyi Biotec, Bergisch-Gladbach, Germany); VioBlue anti-CD3 (clone: 17A2; Miltenyi Biotec, Bergisch-Gladbach, Germany); PerCP/Cy5.5 anti-CD4 (clone: GK1.4; BioLegend, London, UK); Brilliant Violet 711 anti-CD8 (clone: 53–6.7; BioLegend, London, UK); SuperBright600 anti-CD19 (clone: 1D3; eBioscience, San Diego, USA); Brilliant Violet 650 anti-NK1.1 (clone: PK136; BioLegend, London UK); Brilliant Violet 785 anti-CD11b (clone: M1/70; BioLegend, London, UK); Alexa Fluor 488 anti-CD11c (clone: N418; BioLegend, London, UK); PE anti F4/80 (clone: REA126; Miltenyi Biotec, Bergisch-Gladbach, Germany); PE anti-MerTK (clone: REA477; Miltenyi Biotec, Bergisch-Gladbach, Germany); Alexa Fluor 647 anti-SiglecF (clone: S17007L; BioLegend, London, UK); APC-Fire810 anti-Ly6G (clone: 1A8; BioLegend, London, UK) and APC-Cy7 anti-CD127 (clone: A7R34; BioLegend, London, UK) for 20 min at 4°C in FACS buffer (PBS with 0.5% FCS) in the dark. The cells were fixed and permeabilized using the Transcription Factor Staining Buffer Set (Miltenyi Biotec, Bergisch-Gladbach, Germany) prior to staining of the intracellular antibodies. Afterwards the cells were incubated for 30 min in the dark in a mixture of intracellular marker antibodies: Brilliant Violet 421 anti-FoxP3 (clone: MF-14; BioLegend, London, UK); APC anti-RORγt (clone: REA278; Miltenyi Biotec, Bergisch-Gladbach, Germany) and PE-Cy7 anti-T-bet (clone: 4B10; BioLegend, London, UK). The samples were measured by Cytek Aurora (Cytek Bioscience, Fremont, CA, USA) using SpectroFlow (v. 2.2.0.3) and analyzed in FlowJo (v10.0.7, Tree Star Inc., San Carlos, CA, USA). Cell populations of interest were characterized as follows: dendritic cells, CD45^+^ CD11b^+^ CD11c^+^; neutrophils, CD45^+^ CD11b^+^ CD11c^-^ Ly6G^+^; eosinophils, CD45^+^ CD11b^+^ CD11c^-^ SiglecF^+^; macrophages, CD45^+^ CD11b^+^ CD11c^-^ Ly6G^-^ SiglecF^-^ F4/80^+^; natural killer (NK) cells, CD45^+^ CD11b^-^ CD3^-^ NK1.1^+^; natural killer T (NKT) cell, CD45^+^ CD11b^-^ CD3^+^ NK1.1^+^; T cells, CD45^+^ CD11b^-^ NK1.1^-^ CD3^+^; T helper (Th) cells, CD45^+^ CD11b^-^ NK1.1^-^ CD3^+^ CD4^+^; cytotoxic T cells, CD45^+^ CD11b^-^ NK1.1^-^ CD3^+^ CD8^+^; memory and effector Th cells, CD45^+^ CD11b^-^ NK1.1^-^ CD3^+^ CD4^+^ CD127^+^; regulatory T (Treg) cells, CD45^+^ CD11b^-^ NK1.1^-^ CD3^+^ CD4^+^ FoxP3^+^; T-bet positive CD4^+^ cells, CD45^+^ CD11b^-^ NK1.1^-^ CD3^+^ CD4^+^ T-bet^+^; RORγt positive CD4^+^ cells, CD45^+^ CD11b^-^ NK1.1^-^ CD3^+^ CD4^+^ RORγt^+^; B cells, CD45^+^ CD11b^-^ NK1.1^-^ CD3^-^ CD19^+^.

### Statistics

The data were analyzed and visualized by GraphPad Prism (v. 9.5.1). Pre-tests were performed to test the data for homogeneity of variances (Levene’s test) and normal distribution (Shapiro-Wilk test). In the case of homogeneous variances and a normal distribution of the data, one-way ANOVA was used followed by Tukey’s or Dunett’s post hoc test and for heterogeneous variances and/or non-parametric data, Kruskal-Wallis test followed by Dunn’s post hoc test was applied to evaluate differences between the groups as indicated in the figure legends. *P*-values < 0.05 were considered significant and are shown as follows: *p < 0.05, **p < 0.01, ***p < 0.001, (BH versus (vs.) LB vs. PR); ^#^ *p* < 0.05, ^##^ *p* < 0.01, ^###^ *p* < 0.001 (naive vs. infected (BH, LB, PR)). For homogeneous variances and normally distributed data, the presentation was in the form of mean and standard deviation. In cases of heterogeneous variances or non-parametric data, the median and interquartile range were used.

## Results

### Strain-based disparities in *Schistosoma mansoni* worm burden and size

**Fig 2:**
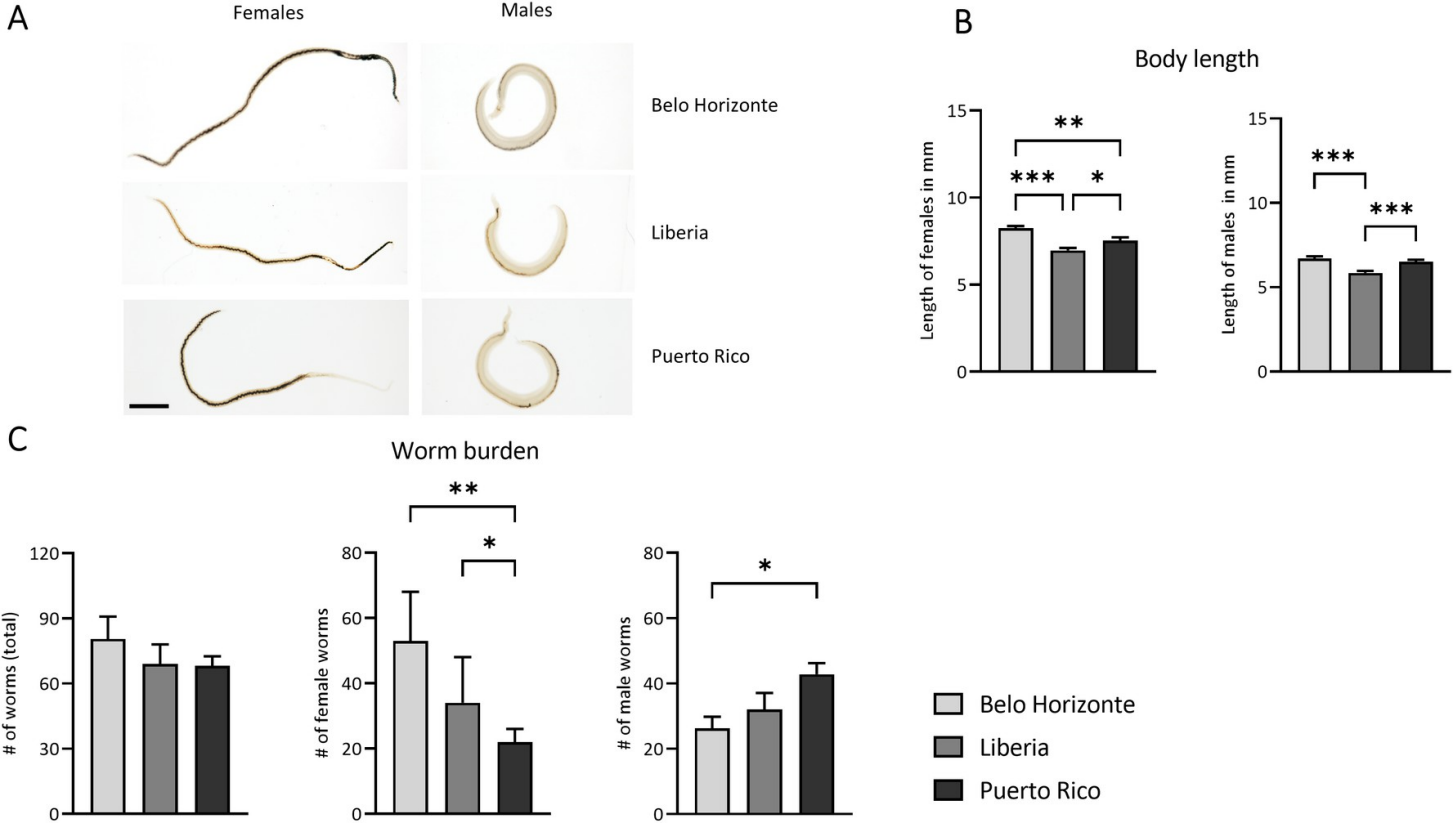
Strain-based disparities in *Schistosoma mansoni* worm burden and size. (A) Representative images of *S*. *mansoni* worm from the respective strains; bar corresponds to 1 mm. (B) Length of adult *S*. *mansoni* worms from the BH, LB and PR strains; worms from the LB strain were significantly smaller than those from the BH or PR strain (C) Worm burden per mouse; the PR strain had significantly fewer female worms, while it had significantly more male worms than the BH strain ((B) n = 21; (C) BH and PR n = 7, LB n = 6). The p-values were obtained either from a one-way ANOVA (length of females, length of males, number of worms (total), number of male worms) with mean and SEM or from a Kruskal-Wallis test with median and interquartile range (number of female worms). Belo Horizonte, BH; Liberia, LB; Puerto Rico, PR. *p*-values < 0.05 were considered statistically significant. * *p* < 0.05, ** *p* < 0.01, *** *p* < 0.001 (BH vs. LB vs. PR); # *p* < 0.05, ## *p* < 0.01, ### *p* < 0.001 (naive vs. infected).

In order to characterize morphological differences and worm burden, the portal venous system of mice infected with 300 cercariae of the respective strain (BH, LB or PR) was perfused 42 dpi. Representative pictures of the respective strains are shown in Fig 2A. Significant differences in body length were observed among the female worms of all strains, with the BH worms being the largest and LB worms being the smallest (mean and SEM of female body length BH: 8.24 ± 0.6; LB 7 ± 0.6; PR: 7.53 ± 0.8). Similar variations were noted in the body length of male adult worms, with the LB strain showing the smallest worms (Fig 2B) (mean and SEM of male body length BH: 6.71 ± 0.13; LB 5.84 ± 0.13 PR: 6.51 ± 0.12). With respect to worm numbers, the BH strain exhibited a significant difference compared to the PR strain, showing the highest number of female worms and the fewest male worms. Conversely, the PR strain displayed the fewest female worms and the highest number of male worms (Fig 2C) (mean and SEM of male worm number BH: 26.3 ± 3.6; LB: 32 ± 5.1; PR: 42.9 ± 3.4; median and interquartile range of female worm number: BH: 53 ± 32; LB: 34 ± 22; PR: 22 ± 5). This shows a difference in the worm sex ratio between the strains.

### Egg characteristics and distribution across *Schistosoma mansoni* strains

Following the analysis of the worm characteristics, the investigation was extended to the characteristics of the eggs and oviposition. The characteristics of eggs laid *in vitro* and those extracted from the liver varied among the strains.

**Fig 3:**
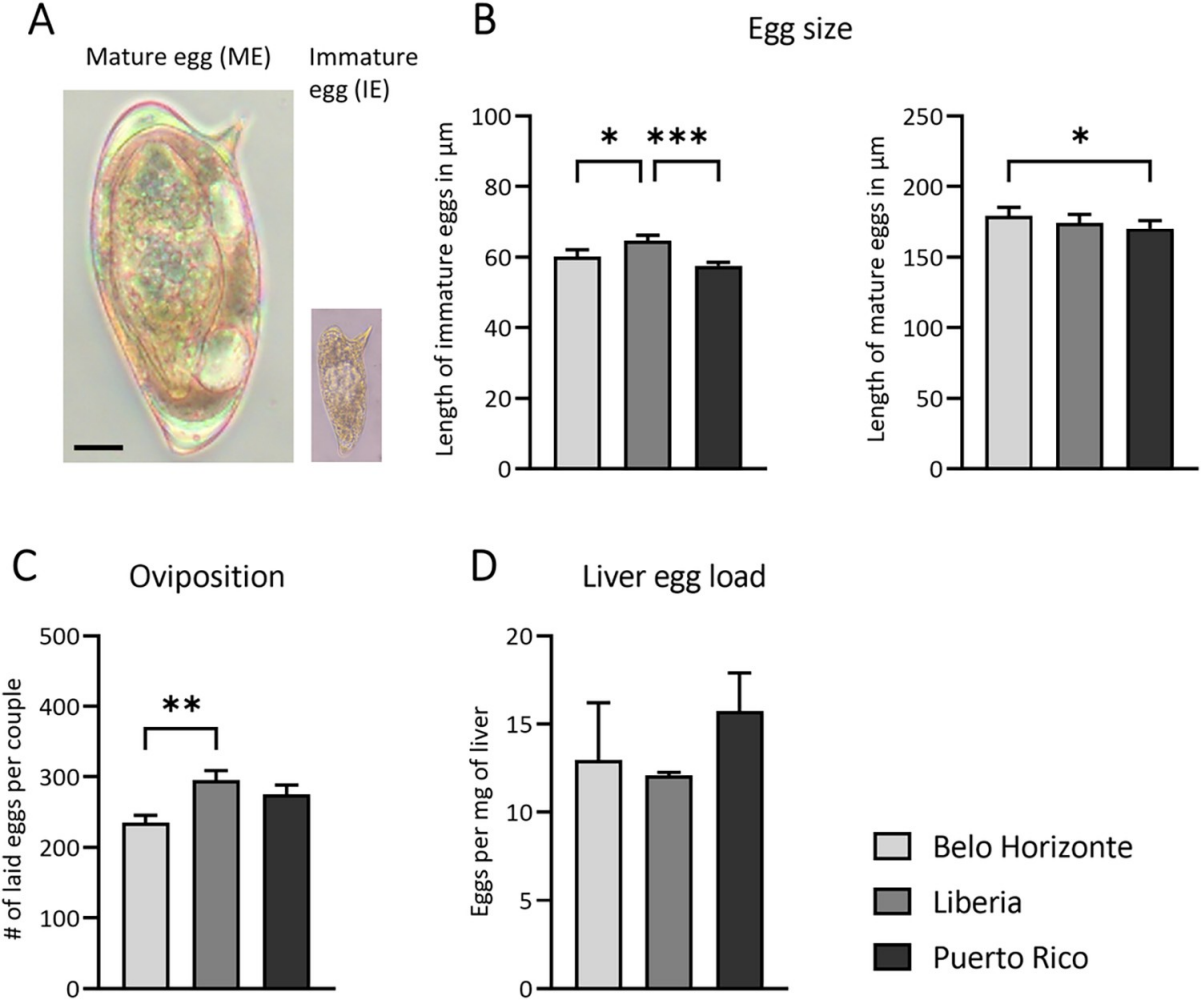
Egg characteristics and distribution across *Schistosoma mansoni* strains. (A) Mature eggs from the liver compared to immature eggs laid in vitro, the bar corresponds to 25 μm. (B) Length of immature *S*. *mansoni* eggs laid *in vitro* and length of mature eggs isolated from liver tissue. The LB strain had the largest immature eggs laid *in vitro*, while the BH strain had the largest mature eggs isolated from liver tissue (C) Eggs laid *in vitro* per worm pair; the LB strain had the most eggs laid *in vitro* per worm pair (D) The egg load in the liver of NMRI mice did not differ between strains. ((B) n = 14; (C) n = 21; (D) BH, PR n = 7, LB n = 6). p-values were obtained from either one-way ANOVA ((C) oviposition) with mean and SEM or Kruskal-Wallis test with median and interquartile range ((A) egg size, (D) liver egg load). *p*-values < 0.05 were considered statistically significant. Belo Horizonte, BH; Liberia, LB; Puerto Rico, PR. * *p* < 0.05, ** *p* < 0.01, *** *p* < 0.001 (BH vs. LB vs. PR); # *p* < 0.05, ## *p* < 0.01, ### *p* < 0.001 (naive vs. infected).

Fig 3A illustrates a representative comparison of the size of mature and immature *in vitro* laid eggs. *In vitro*, the LB strain exhibited the largest immature eggs (Fig 3B) compared to the other strains (median and interquartile range of *in vitro* egg size: BH: 60.20 ± 3.35; LB: 64.65 ± 9.88; PR: 57.50 ± 10.15). Moreover, the LB strain displayed significantly more eggs per worm couple compared to the BH strain (Fig 3C) (mean and SEM of *in vitro* egg ovipositon: BH: 235.2 ± 10.29; LB: 295.3 ± 13.48; PR: 275.2 ± 13.13).

*In vivo*, extracted mature eggs from the liver were largest in the BH strain, showing a significant difference compared to the PR strain, which had the smallest mature eggs (Fig 3B) (median and interquartile range of mature egg size: BH: 179.3 ± 9.1; LB: 174.3 ± 10; PR: 170.3 ± 14.5). Although the PR strain showed the highest egg load in the liver, the differences compared to the LB and BH strains did not reach statistical significance (Fig 3D) (median and interquartile range of egg number per mg liver tissue: BH: 12.95 ± 7.15; LB: 12.07 ± 2.21; PR: 15.74 ± 9.7).

### Infection related impact of different *Schistosoma mansoni* strains on host pathology

**Fig 4:**
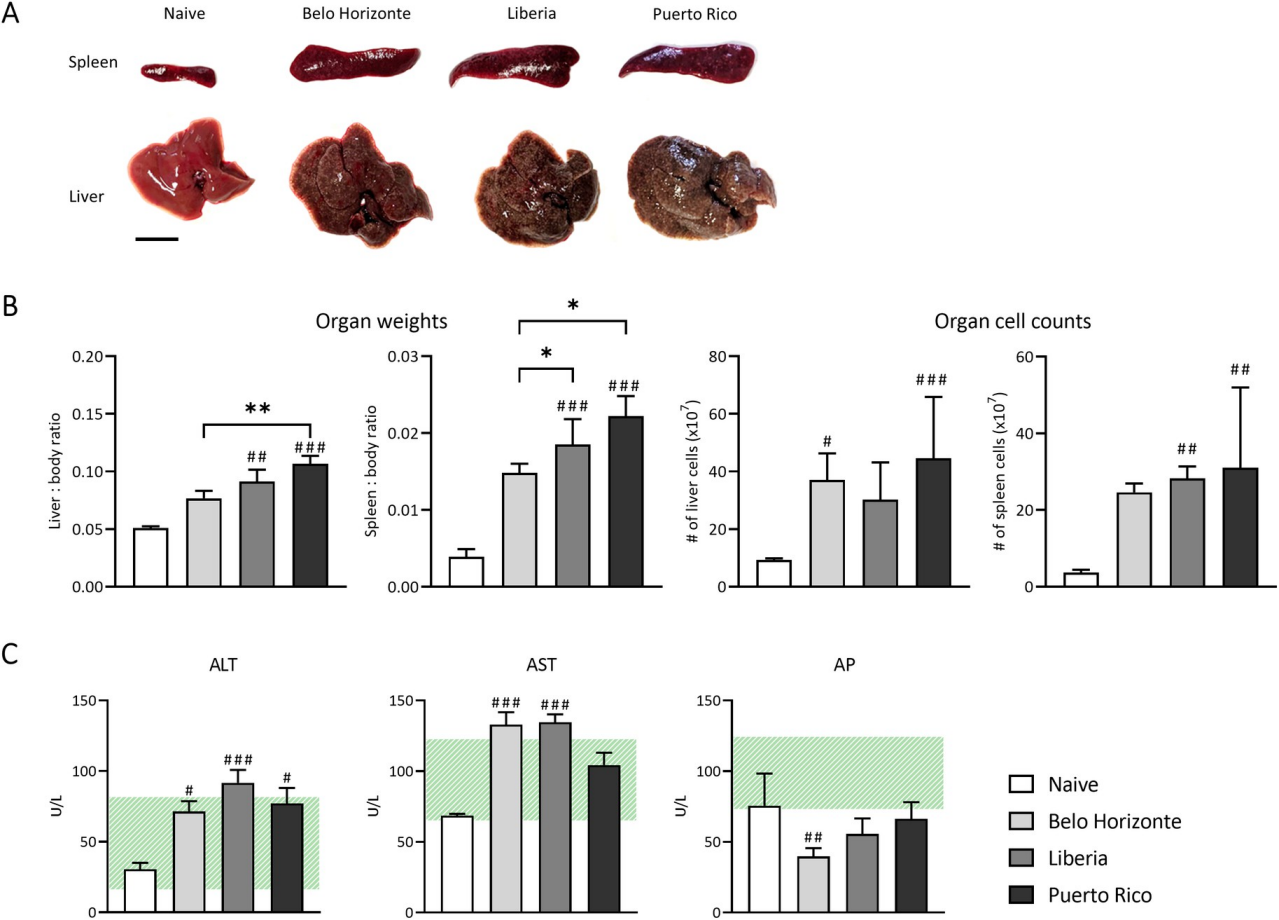
Infection related impact of different *Schistosoma mansoni* strains on host pathology. (A) Liver and spleen pathology of mice infected with 100 cercariae of the BH, LB and PR strain; the bar corresponds to 1 cm. (B) Ratio of liver and spleen weight to body weight and organ cell counts; the PR strain had the highest ratio of organs to body weight, which also tended to be reflected in organ cell counts (D) Liver enzymes alanine aminotransferase (ALT) and aspartate aminotransferase (AST) were elevated during infection with all three strains, with the exception of AST in the PR group. Alkaline phosphatase (AP) tended to be decreased in all 3 infection groups and reached mathematical significance in the BH group. The range of normal blood parameters of 10-week-old RjHan:NMRI mice from Janvier Labs is shaded green (https://janvier-labs.com/en/fiche_produit/nmri_mouse/). (Naive, BH and PR n = 7, LB n = 6). p-values were obtained from either one-way ANOVA ((D): BH vs. LB vs. PR) with mean and SEM or from Kruskal-Wallis test with median and interquartile range ((B), (C) and (D): BH, LB, PR vs. N). *p*-values < 0.05 were considered statistically significant. Belo Horizonte, BH; Liberia, LB; Puerto Rico, PR. * *p* < 0.05, ** *p* < 0.01, *** *p* < 0.001 (BH vs. LB vs. PR); # *p* < 0.05, ## *p* < 0.01, ### *p* < 0.001 (naive vs. infected).

After describing the *S*. *mansoni* characteristics, we investigated the intra-specific pathological effects on the liver and spleen of the host (Fig 4A). Mice infected with the LB and PR strains had significantly higher liver/body weight and spleen/body weight ratios compared to uninfected mice (Fig 4B). In particular, infection with the PR strain resulted in an even higher liver-to-body weight ratio and heavier livers than in mice infected with the BH strain. In addition, both the PR and LB strains caused a significantly higher spleen/body weight ratio than infection with the BH strain (Fig 4B). There was no statistically significant difference in liver or spleen cell count between infection groups.

All three *S*. *mansoni* strains showed elevated levels of liver transaminases after infection compared to the healthy control group (Fig 4C), although even the highest values deviate only slightly from the normal values of a healthy mouse (hatched area in Fig 4C).

### Liver pathology variation induced by different *Schistosoma mansoni* strains

**Fig 5:**
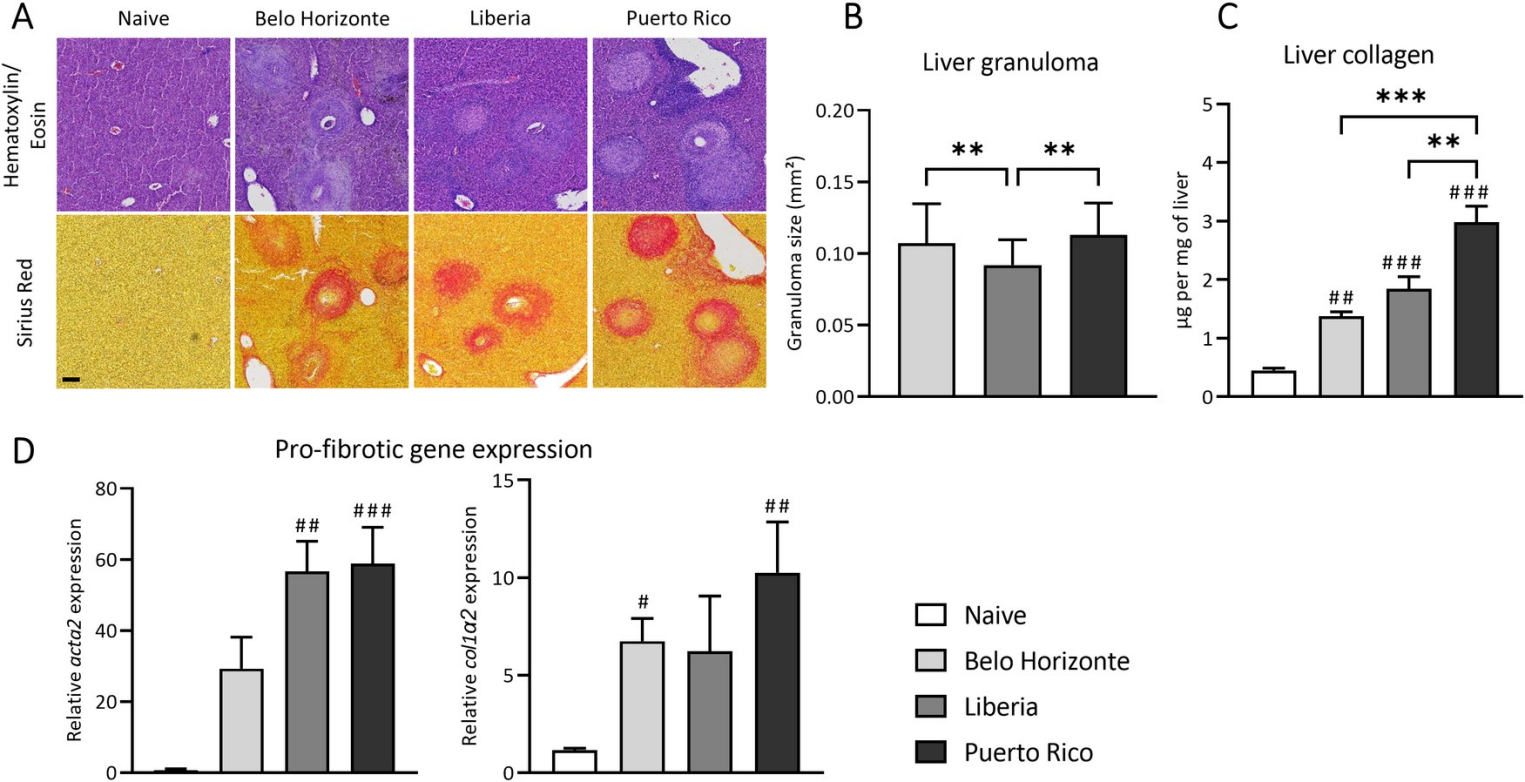
Liver pathology variation induced by different *Schistosoma mansoni* strains. (A) H&E and SR staining of liver granulomas from mice infected with BH, LB and PR strain; the bar represent 100 μm (B) The LB group showed smaller granulomas than the BH and PR groups (C) Liver collagen content was highest in the PR group (D) Gene expression of *acta2* and *col1α2* tended to be increased in all infection groups, but the highest expression was found in the PR group. ((A), (B), BH and PR n = 70, LB n = 60; (C) and (D): Naive, BH and PR n = 7, LB n = 6). The values were either analyzed by a one-way ANOVA ((C); (D): (BH vs. LB vs. PR) with mean and SEM or by a Kruskal-Wallis test with median and interquartile range ((B), (C): (BH, LB, PR vs. N) and (D): BH, LB, PR vs. N) were determined. Belo Horizonte, BH; Liberia, LB; Puerto Rico, PR. *p*-values < 0.05 were considered statistically significant. * *p* < 0.05, ** *p* < 0.01, *** *p* < 0.001 (BH vs. LB vs. PR); # *p* < 0.05, ## *p* < 0.01, ### *p* < 0.001 (naive vs. infected).

The differences in liver pathology between hosts infected with the respective *S*. *mansoni* strains, prompted us to extend our research to analyze fibrotic response in the liver. The size of hepatic granulomas caused by the eggs of the LB strain was significantly smaller than those induced by eggs from the PR or BH strain (Fig 5A and 5B) (median and interquartile range of granuloma size: BH: 0.1073 ± 0.048; LB: 0.092 ± 0.038; PR: 0.1 ± 0.05). In contrast, mice infected with the PR strain exhibited significantly more liver collagen compared to the other strains (Fig 5C) (mean and SEM of collagen content in μg per mg liver tissue: naive: 0.453 ± 0.04; BH: 1,4 ± 0.08; LB: 1.8 ± 0.21; PR: 2.98 ± 0.3). The qPCR analysis addressing the expression of pro-fibrotic genes, supported this observation, showing increased *col1α2* and *acta2* relative gene expression in the PR group, although not reaching mathematical significance between infected groups (Fig 5D).

### Distinct inflammatory responses elicited by strain-specific *Schistosoma mansoni* infections

**Fig 6:**
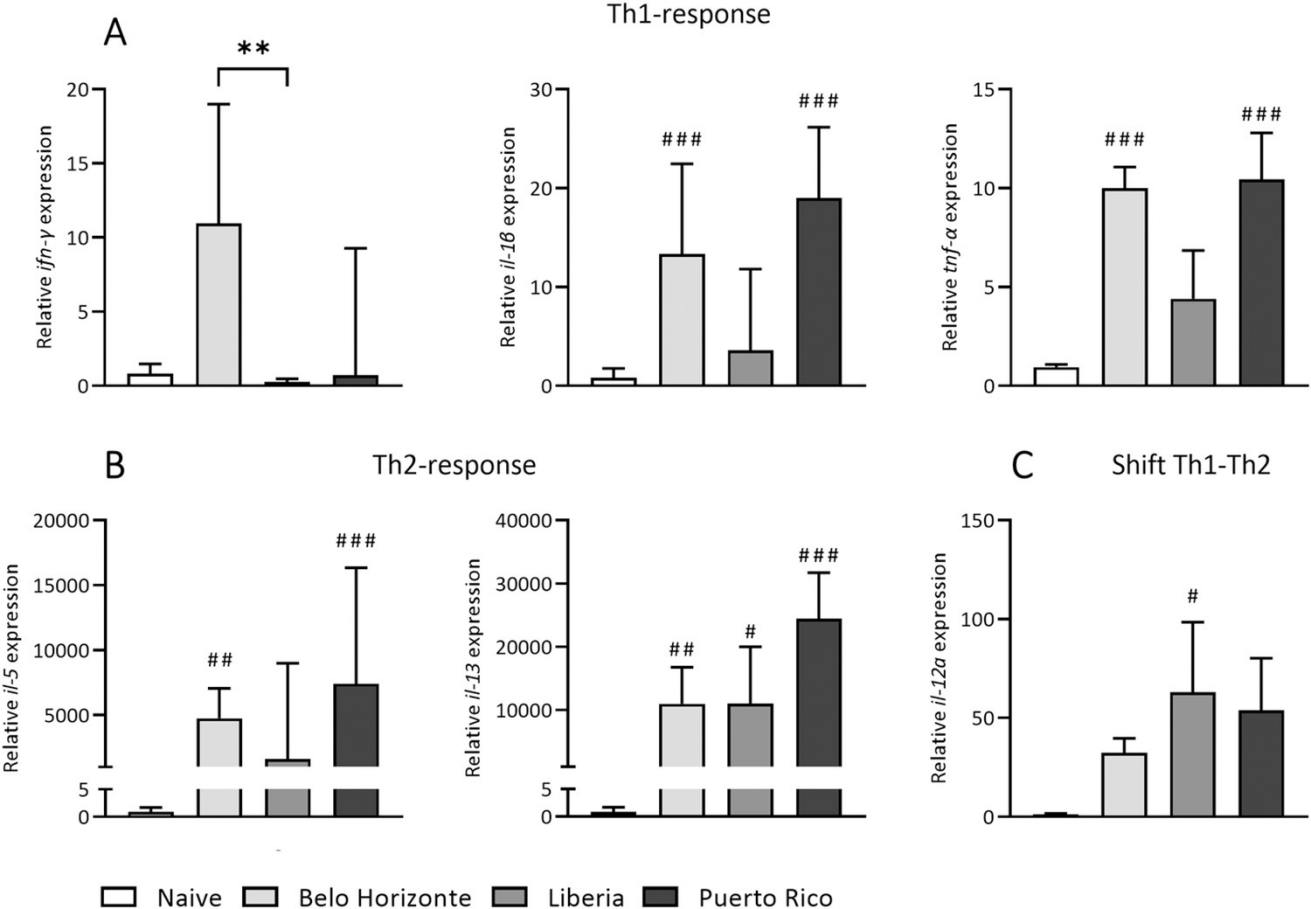
Distinct inflammatory responses elicited by strain-specific *Schistosoma mansoni* infections. The relative gene expression of Th1 cytokines was lowest in the LB group, while the PR group had the highest expression of Th2 cytokines. (A) Th1 cytokine gene expression, *ifn-γ*, *il-1β* and *tnf-α* (B) Th2 cytokine gene expression, *il-5* and *il-13* (C) Th1-Th2 shift, *il-12a* gene expression. (Naive, BH and PR n = 7, LB n = 6) Values resulted either from one-way ANOVA (*tnf-α*: BH vs. LB vs. PR) with mean and SEM or from Kruskal–Wallis test with median and interquartile range (*ifn-γ*, *il-1β*, *il-5*, *il-13* and *il-12a*; *tnf-α*: BH, LB, PR vs. N). *p* values < 0.05 were considered statistically significant. Belo Horizonte, BH; Liberia, LB; Puerto Rico, PR. * *p* < 0.05, ** *p* < 0.01, *** *p* < 0.001 (BH vs. LB vs. PR); # *p* < 0.05, ## *p* < 0.01, ### *p* < 0.001 (naive vs. infected).

We analyzed the gene expression levels of Th1 and Th2 inflammatory markers. The BH group showed a significant increase in *ifn-γ* compared to the LB group (median and interquartile range of *ifn-γ*: BH: 11 ± 11.3; LB: 0.3 ± 0.3). Relative expressions of *il-1β* and *tnf-α* in mice infected with the BH and PR strains showed marked elevation compared to uninfected animals. The LB group tended to exhibit the lowest Th1 response (Fig 6A) but showed a significant increase in *il-12a* expression compared to uninfected mice (Fig 6C). There was a significant (not for *il-5* in the LB group) increase in the expression of *il-5* and *il-13* in all three infection groups compared to uninfected mice, with the PR group reaching the highest levels (Fig 6B).

### Immunological alterations in the liver due to different *Schistosoma mansoni* strains

**Fig 7:**
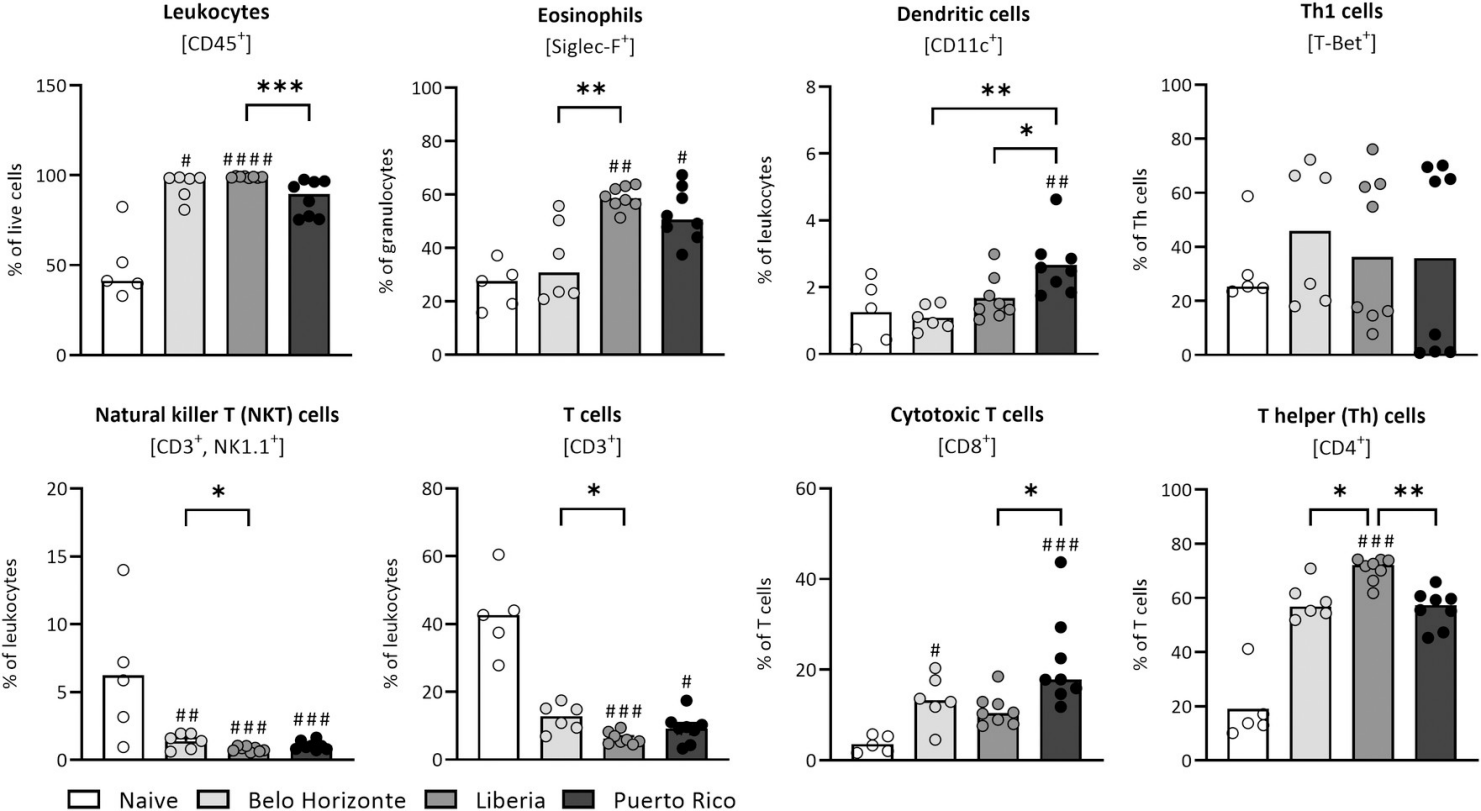
Immunological alterations in the liver due to infection with different *Schistosoma mansoni* strains. Flow cytometric analysis of liver samples from mice infected with the BH, LB and PR *S*. *mansoni* strains revealed different immune responses between the strains. The Leukocytes as % of live cells, dendritic cells and natural killer T cells as % of leukocytes, eosinophils as % of granulocytes, cytotoxic T cells and T helper cells as % of T cells and Th1 cells as % of T helper cells. Naive, BH and PR n = 7, LB n = 6. Values resulted either from one-way ANOVA (dendritic cells and natural killer T cells; cytotoxic T cells: BH vs. LB vs. PR) with mean or from Kruskal–Wallis test with median (leukocytes, eosinophils, T cells, T helper cells, Th1 cells; cytotoxic T cells BH, LB, PR vs. N). *p* values < 0.05 were considered statistically significant. Belo Horizonte, BH; Liberia, LB; Puerto Rico, PR. * *p* < 0.05, ** *p* < 0.01, *** *p* < 0.001 (BH vs. LB vs. PR); # *p* < 0.05, ## *p* < 0.01, ### *p* < 0.001 (naive vs. infected).

We performed a flow cytometry analysis of the liver samples. The mice infected with the LB strain had significantly more leukocytes (percentage of live cells) in the livers than those infected with the PR strain (Fig 7) (median of leukocytes: LB: 99; PR: 89.6). In addition, infection with the LB strain resulted in significantly more eosinophils (percentage of granulocytes) than infection with the BH strain (eosinophil median: BH: 30.8; LB: 58.7). Further, infection with the LB strain resulted in a significant decrease in natural killer T cells (as a percentage of leukocytes) and T cells (as a percentage of leukocytes), compared with the BH-infected group (natural killer T cells median: BH: 1.5; LB: 0.8; T cell mean: BH: 12.4; LB: 6;2). Moreover, the LB group showed significantly fewer cytotoxic T cells than the PR group (cytotoxic T cells mean: LB: 11.2; PR: 21.7). The T helper cells of the LB group were significantly increased compared to the PR and BH groups (median of T helper cells: BH: 57; LB: 72; PR: 57.4). Mice infected with the PR strain had significantly more dendritic cells (percentage of leukocytes) than all other groups (Fig 7) (mean of dendritic cells: BH: 1.1; LB: 1.67; PR: 2.7).

### Strain-dependent immunological changes in the spleen following *Schistosoma mansoni* infection

**Fig 8:**
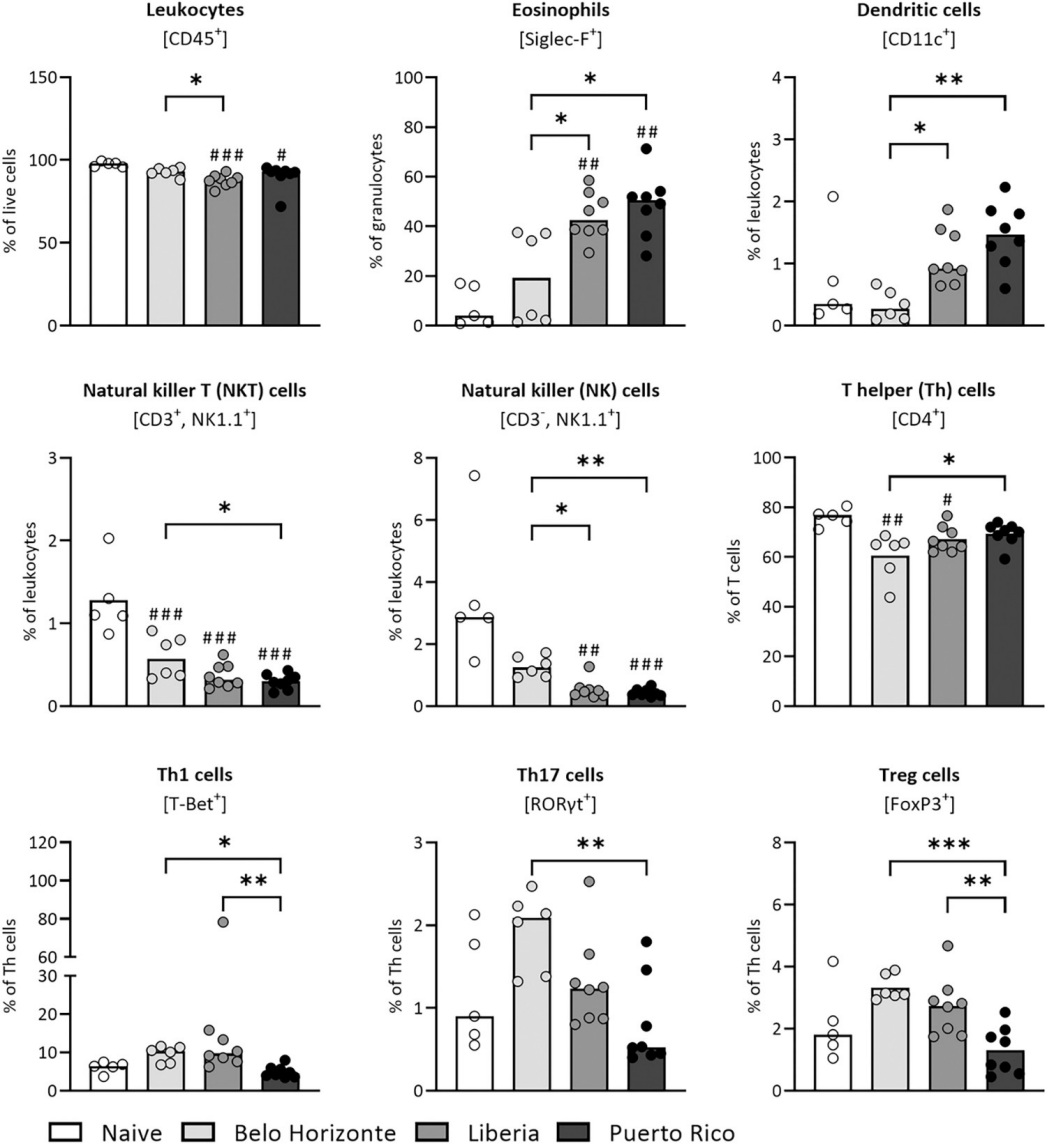
Strain-dependent immunological changes in the spleen following *Schistosoma mansoni* infection. Flow cytometric analysis of spleen samples from mice revealed distinct immune responses in the BH, LB and PR *S*. *mansoni* strains. Leukocytes as % of live cells, dendritic cells and natural killer T cells as % of leukocytes, eosinophils as % of granulocytes, helper T cells as % of T cells, Th1 cells, Th17 cells and Treg cells as % of T helper cells. (Naive, BH and PR n = 7, LB n = 6) Values resulted either from one-way ANOVA (natural killer T cells: BH, LB, PR vs. N, Th cells and Treg cells: BH vs. LB vs. PR) with mean or from Kruskal–Wallis test with median (leukocytes, eosinophils, dendritic, Th1 cells, Th17 cells; Th cells and regulatory T cells: BH, LB, PR vs. N; natural killer T cells: BH vs. LB vs. PR). Belo Horizonte, BH; Liberia, LB; Puerto Rico, PR. *p* values < 0.05 were considered statistically significant. * *p* < 0.05, ** *p* < 0.01, *** *p* < 0.001 (BH vs. LB vs. PR); # *p* < 0.05, ## *p* < 0.01, ### *p* < 0.001 (naive vs. infected).

We also used flow cytometry to assess differences in cellular immune responses in the spleen. Mice infected with the LB had significantly fewer leukocytes (percent of live cells) in the spleen compared to the BH group (Fig 8) (median of leukocytes: BH: 93; LB: 88). Both the LB and PR groups had significantly more eosinophils (percentage of granulocytes) and dendritic cells (percentage of leukocytes) in the spleen than the BH-infected group (median of eosinophils: BH: 19; LB: 43; PR: 51; median of dendritic cells: BH: 0.3; LB: 0.92; PR: 1.5). The PR group had significantly fewer natural killer T cells (as a percentage of leukocytes) than the BH group (Fig 8) (median of natural killer T cells: BH: 0.6; PR: 0.3). The BH group showed significantly more natural killer cells (as a percentage of leukocytes) than the LB and PR groups and significantly more Th17 cells (percentage of helper T cells) than the PR group (Fig 8) (median of natural killer cells: BH: 1.3; LB: 0.5; PR: 0.4; median of Th17 cells: BH: 2.1; PR: 0.5). However, the BH group showed significantly fewer T helper cells (percentage of T cells) than the PR group (Fig 8) (mean of T helper cells: BH: 61; PR: 69.3). The mice infected with PR had significantly fewer Th1 cells and regulatory T cells (percentage of T helper cells) than the LB and BH groups (Fig 8) (median of Th1 cells: BH: 10; LB: 9.7; PR: 4.5; mean of regulatory T cells: BH: 3.3; LB: 2.73; PR: 1.3).

## Discussion

Our comprehensive study of three different *S*. *mansoni* laboratory strains from Belo Horizonte, Liberia and Puerto Rico revealed considerable intraspecific variability, in particular with respect to virulence, host-parasite interactions and immune responses. We found differences in worm sex distribution, granuloma and fibrosis formation and their impact on host organ pathology. Infection with the PR strain tended to be associated with higher egg loads and more severe organ lesions, in contrast, infection with the LB strain tended to have fewer egg loads and showed less liver fibrosis, suggesting a less pathogenic profile. These results emphasize the complexity of schistosomiasis and the importance of intra-specific differences. Our study also sheds light on the interaction between the host immune response and the parasite, as evidenced by different patterns in granuloma formation, T cell dynamics and cytokine levels in the defined mouse model.

An important distinctive feature in the pathogenesis of schistosomiasis is the pattern of egg distribution in the tissue. The PR infected group, while exhibiting a similar egg density per milligram of liver tissue as the BH infected group, displayed significantly heavier liver to bodyweight ratios. This indicates a higher overall egg count in the PR group. Consistent with existing literature, it is known that studied African strains, such as the LB strain, produce fewer and smaller eggs [27,31,42]. In contrast, American strains, exemplified by our findings on the PR strain, tend to show higher egg quantities in host organ tissues [27–30, 32]. The consensus in schistosomiasis research suggests that the severity of the disease is correlated with the presence and burden of eggs [43]. In infections caused by *S*. *mansoni* and *S*. *japonicum*, the eggs trapped in the host organs, primarily in the liver, lead to the formation of granulomas. These granulomas play an essential role in the progression of the disease [44]. Our data suggest that the PR group, with its heavier spleen and liver weights coupled with higher egg loads, indicates more severe liver pathology. The increased organ weight in this group could be attributed to enhanced liver fibrosis, as shown by the increased collagen content. Our research clearly shows that the parasite strain significantly influences the pathology of the host. However, it is known that host genetics also play a crucial role in the outcome of the infection [29], particularly through the Th1 response, including the production of IFN-γ and TNF-α [45].

With regard to the worm-sex-ratio, a significant difference was observed in the female-to-male worm ratio between the PR and BH strains. The former showed a lower ratio, while the latter had a higher prevalence of female worms. Our own research has emphasized the immunosuppressive effects of female worms, such as smaller perioval granulomas and reduced hepatic fibrosis, resulting in a lower organ-to-body weight ratio [46, 47]. Therefore, the pronounced organ changes in the PR group may be linked to a higher male worm prevalence. Further complicating the picture, our latest study reveals distinct immune responses to male, female, and bisexual infections at the lung stage. This raises questions about the impact of worm sex ratios on the host [48]. The higher female-to-male worm ratio observed in the BH group may correlate with lower eosinophil levels in the liver and spleen. Female worms are known to suppress the innate immune response [47,49]. Eosinophils, as vital elements of the innate immune system, combat schistosomes through the release of hydrogen peroxide [50] and play a significant role in granuloma formation during schistosome infections.

Our study sheds light on the complicated pathology of schistosomiasis, in particular on the balance between Th1 and Th2 responses and the involvement of Th17 cells. The PR group exhibited the highest gene expression levels of Th2 cytokines, corresponding with a lower Th1 cell count in the spleen and indicating a dominant Th2 response. In contrast, the LB strain had the highest Th1 cell count in the spleen, although this was not entirely consistent with the qPCR results for Th1 cytokines, which indicated the lowest levels in this group. However, the gene expression of Th1 cytokines was significantly lower than that of Th2 cytokines, indicating a predominant Th2 response in all strains.

The BH group showed a unique profile with the highest count of Th17 cells, known for IL-17 production. IL-17’s role in initiating a broad inflammatory response considerably contributes to the disease’s pathology, marked by increased inflammation and tissue damage [51]. In Schistosomiasis and other liver diseases, elevated levels of Th1 and Th17 are often linked to increased inflammation [52,53], which could explain the tendentially higher liver enzyme levels observed in both the BH and LB groups. Interestingly, it was the PR group that displayed the most severe pathological outcomes, despite showing the lowest Th1 and Th17 responses among the strains. The severity in the PR group may be attributed to its pronounced Th2 response, leading to significantly higher collagen content in the liver, indicative of greater fibrosis. This aspect of the disease has been extensively reviewed in the literature [54]. In addition, the severity, higher Th2 response and higher degree of fibrosis in the PR infection group could be due to an earlier onset of oviposition than in the other groups, which in turn would lead to an earlier Th1/Th2 shift in this infection group. Analyses of the course of infection of the three respective strains, including worm burden and egg laying, are already underway.

The heightened Th2 response in the PR group could be linked to increased dendritic cell counts, particularly in the liver and comparably high levels in the spleen, as observed in the LB group. Dendritic cells can produce strong Th2 responses, when soluble egg antigens are present [55,56]. This may contribute to the enhanced Th2 cytokine expression, especially IL-13, a cytokine critically involved in fibrosis [57–59], likely influenced by the egg glycoprotein Omega-1, which directs dendritic cells towards Th2 cell expansion [60]. Interestingly, the differences in liver collagen content did not correspond to granuloma size. The LB group, with a similar collagen content to the BH group, uniquely presented smaller granulomas. Conversely, the PR group, despite having the highest liver collagen content, exhibited granulomas similar in size to those of the BH group. We hypothesise that the PR strain causes a more scarring disease course, characterised by less progressive destruction of hepatocytes at the time of measurement, as the PR group did not show elevated AST levels compared to the naive mice. Granulomas play a crucial role for the host and the parasite. They are key for egg transmigration through the intestinal barrier. At the same time, they protect the host’s tissue by encapsulating hepatotoxic secretions. The granuloma size usually decreases 8 to 20 weeks after infection due to increasing immunohypersensitivity [61]. The smaller granulomas in the LB group may indicate an earlier onset of immune hyporesponsiveness, suggested by their lower Th1 cytokine level. However, a corresponding decrease in Th2 cytokine levels was not observed [6]. The differences in granuloma size and collagen content across strains underscore variations in immune regulation and fibrosis, suggesting the host’s immune response plays a crucial role in fibrotic outcomes, independent of granuloma size [62].

T-cells play a central role in the formation of granulomas during schistosomiasis. Our analysis shows that infection with the LB strain results in a significant reduction in total T-cell numbers compared to mice infected with the BH strain. The LB group has the highest relative prevalence of CD4^+^ T cells, alongside smaller hepatic granulomas. This suggests that mice infected with the LB strain possess lower absolute levels of CD4^+^ cells. The depletion of CD4^+^ cells is associated with the formation of smaller granulomas, but could exacerbate disease severity [63,64]. Another critical factor in determining granuloma size and disease severity is IFN-γ. In general, high levels of IFN-γ interfere with granuloma formation and lead to a reduction in granuloma size [65,66]. However, this is in contrast to the BH-infected group, which, despite having the highest *ifn-γ* expression levels, particularly in comparison to the negligible levels in the LB group, exhibits larger granulomas. The elevated *ifn-γ* expression levels in the BH group may be due to an increased presence of NK-Cells [67]. IFN-γ is essential in the innate immune response against schistosomes, promoting the expression of classically activated macrophages [68], stimulating a Th1 response and inhibiting a Th2 response [69]. IFN-y is one of the most potent antifibrotic cytokines in schistosome egg induced liver fibrosis [70], possibly explaining the lowest levels of fibrosis in the BH group.

In concluding our comparative analysis of *S*. *mansoni* strains from Belo Horizonte, Liberia and Puerto Rico during mouse infection, we have gained important insight into the intricate nature of schistosomiasis. This disease, with far-reaching public health implications, exhibits considerable intra-specific variability in *S*. *mansoni*. Our study reveals distinct patterns of virulence, host-parasite interactions, immune responses, and organ pathology. Notably, the PR strain, with its severe fibrosis, extensive organ alterations, high egg loads, and elevated male-to-female worm ratios, emerges as the most potentially virulent. Yet, further analyses are required to fully elucidate the complex host-parasite dynamics and the nuances therein.

The limitations of our study concern the strain-specific differences of the geographically distinct *S*. *mansoni* strains, which have not yet been sufficiently characterized. The rate of development and onset of oviposition could differ between strains, which could influence granuloma size as well as cellular responses, inflammatory processes, and granuloma formation. This is currently being clarified in a more detailed study. Another potential limitation of our study is that we infected the mice with cercariae from bisexually infected snails. This means that we cannot accurately estimate the sex ratio of the cercariae that we use for infection and therefore cannot precisely predict how many worm pairs will ultimately be present in the mouse. An alternative would have been to infect the snails unisexually and use an exact mix of 50 female and 50 male cercariae for mouse infection. However, this does not solve the question of how many worm pairs are ultimately present. This is because the different infectivity of male and female cercariae must be taken into account [71,72], which is a nuanced and complex task and certainly also depends on the mode of infection (water bath or injection). Furthermore, it is questionable whether models for unisexual infections can be used effectively to determine the infectivity of male or female cercariae, as the development of the parasite differs considerably in unisexual and mixed-sex infections [48,72,73]. A predictable number of adult worm pairs is therefore not possible. In this study, the focus was therefore placed on standardized conditions, so that it can be assumed that each mouse was infected with the same cercariae mix under the same conditions. A further limitation of the study is the use of *S*. *mansoni* strains, which have been kept in the laboratory for decades. We point out that these laboratory strains are likely to behave differently from fresh isolates from the wild. We also chose to use outbred mice, which have greater genetic diversity to better represent the overall population diversity. However, this could lead to a certain heterogeneity in the host immune response.

The differences observed in our study in terms of clinical course and virulence are of crucial importance for a better understanding of the course of the disease and should help to improve therapeutic measures in the future. Although this is an experimental model in mice and the results cannot be transferred one-to-one to humans, the experiments form the basis for subsequent studies to investigate whether differences in immunological responses are due to immunological excretory-secretory (E-S) products of schistosome eggs. The present study thus paves the way for the development of new therapeutic measures or approaches for vaccines to contribute to the control of schistosomiasis.

## Supporting information

S1 Table Minimal Data Set for Fig 2. (A) The worm length (in mm) of male and female adult schistosomes of the three *Schistosoma mansoni* strains from Belo Horizonte (BH), Liberia (LB) and Puerto Rico (PR) is shown. (B) Shown is the number of adult *Schistosoma mansoni* isolated by perfusion of the portal venous system 42 days after infection with 300 cercariae in three experimental groups: infected with Schistosoma mansoni from Belo Horizonte, Liberia and Puerto Rico. (C) Shown is the number of adult *Schistosoma mansoni* isolated by perfusion of the portal venous system 42 days after infection with 300 cercariae of the strain from Belo Horizonte differentiated into male, female and juvenile worms. (D) Shown is the number of adult *Schistosoma mansoni* isolated by perfusion of the portal venous system 42 days after infection with 300 cercariae of the strain from Liberia differentiated into male, female and juvenile worms. (E) Shown is the number of adult *Schistosoma mansoni* isolated by perfusion of the portal venous system 42 days after infection with 300 cercariae of the strain from Puerto Rico differentiated into male, female and juvenile worms.
(XLSX)

S2 Table Minimal Data Set for Fig 3. (A) The length of immature *Schistosoma mansoni* eggs (in μm) is shown in comparison between the Belo Horizonte, Liberia and Puerto Rico strains. (B) The length of mature *Schistosoma mansoni* eggs (in μm) is shown in comparison between the Belo Horizonte, Liberia and Puerto Rico strains. (C) The number of eggs laid in vitro per *Schistosoma mansoni* worm pair is shown in comparison of the strains Belo Horizonte, Liberia, Puerto Rico. (D) The liver egg load is shown in comparison of the Belo Horizonte, Liberia and Puerto Rico *Schistosoma mansoni* strains.
(XLSX)

S3 Table Minimal Data Set for Fig 4. (A) Liver pathology of mice infected with 100 cercariae of *Schistosoma mansoni* strains from Belo Horizonte, Liberia and Puerto Rico: Liver cell count. (B) Liver pathology of mice infected with 100 cercariae of *Schistosoma mansoni* strains from Belo Horizonte, Liberia and Puerto Rico: Spleen cell count. (C) Liver pathology of mice infected with 100 cercariae of *Schistosoma mansoni* strains from Belo Horizonte, Liberia and Puerto Rico: Serum biochemistry, alanine aminotransferase. (D) Liver pathology of mice infected with 100 cercariae of *Schistosoma mansoni* strains from Belo Horizonte, Liberia and Puerto Rico: Serum biochemistry, aspartate aminotransferase. (E) Liver pathology of mice infected with 100 cercariae of *Schistosoma mansoni* strains from Belo Horizonte, Liberia and Puerto Rico: Serum biochemistry, alkaline phosphatase. (F) Liver pathology of mice infected with 100 cercariae of *Schistosoma mansoni* strains from Belo Horizonte, Liberia and Puerto Rico: Spleen to body weight ratio. (G) Liver pathology of mice infected with 100 cercariae of *Schistosoma mansoni* strains from Belo Horizonte, Liberia and Puerto Rico: Liver to body weight ratio.
(XLSX)

S4 Table Minimal Data Set for Fig 5. (A) Liver pathology of mice infected with 100 cercariae of *Schistosoma mansoni* strains from Belo Horizonte, Liberia and Puerto Rico: Liver granuloma size. (B) Liver pathology of mice infected with 100 cercariae of *Schistosoma mansoni* strains from Belo Horizonte, Liberia and Puerto Rico: Hepatic collagen content. (C) Liver pathology of mice infected with 100 cercariae of *Schistosoma mansoni* strains from Belo Horizonte, Liberia and Puerto Rico: Gene expression acta2. (D) Liver pathology of mice infected with 100 cercariae of *Schistosoma mansoni* strains from Belo Horizonte, Liberia and Puerto Rico: Gene expression col1a2.
(XLSX)

S5 Table Minimal Data Set for Fig 6. (A) Liver pathology of mice infected with 100 cercariae of *Schistosoma mansoni* strains from Belo Horizonte, Liberia and Puerto Rico: Gene expression ifn gamma. (B) Liver pathology of mice infected with 100 cercariae of *Schistosoma mansoni* strains from Belo Horizonte, Liberia and Puerto Rico: Gene expression interleukin 1 beta. (C) Liver pathology of mice infected with 100 cercariae of Schistosoma mansoni strains from Belo Horizonte, Liberia and Puerto Rico: Gene expression tnf alpha. (D) Liver pathology of mice infected with 100 cercariae of *Schistosoma mansoni* strains from Belo Horizonte, Liberia and Puerto Rico: Gene expression interleukin 12 alpha. (E) Liver pathology of mice infected with 100 cercariae of *Schistosoma mansoni* strains from Belo Horizonte, Liberia and Puerto Rico: Gene expression interleukin 5. (F) Liver pathology of mice infected with 100 cercariae of *Schistosoma mansoni* strains from Belo Horizonte, Liberia and Puerto Rico: Gene expression interleukin 13.
(XLSX)

S6 Table Minimal Data Set for Fig 7. (A) Flow cytometric analysis of liver samples from mice infected with *Schistosoma mansoni* strain from Belo Horizonte, Liberia and Puerto Rico: Liver leukocytes. (B) Flow cytometric analysis of liver samples from mice infected with *Schistosoma mansoni* strain from Belo Horizonte, Liberia and Puerto Rico: Liver eosinophils. (C) Flow cytometric analysis of liver samples from mice infected with *Schistosoma mansoni* strain from Belo Horizonte, Liberia and Puerto Rico: Liver dendritic cells. (D) Flow cytometric analysis of liver samples from mice infected with *Schistosoma mansoni* strain from Belo Horizonte, Liberia and Puerto Rico: Liver T-helper cells subtype 1. (E) Flow cytometric analysis of liver samples from mice infected with *Schistosoma mansoni* strain from Belo Horizonte, Liberia and Puerto Rico: Liver natural killer cells. (F) Flow cytometric analysis of liver samples from mice infected with *Schistosoma mansoni* strain from Belo Horizonte, Liberia and Puerto Rico: Liver CD8 positive cells. (G) Flow cytometric analysis of liver samples from mice infected with *Schistosoma mansoni* strain from Belo Horizonte, Liberia and Puerto Rico: Liver T-helper cells. (I) Flow cytometric analysis of liver samples from mice infected with *Schistosoma mansoni* strain from Belo Horizonte, Liberia and Puerto Rico: Liver T-cells.
(XLSX)

S7 Table Minimal Data Set for Fig 8. (A) Flow cytometric analysis of spleen samples from mice infected with *Schistosoma mansoni* strain from Belo Horizonte, Liberia and Puerto Rico: Spleen leukocytes. (B) Flow cytometric analysis of spleen samples from mice infected with *Schistosoma mansoni* strain from Belo Horizonte, Liberia and Puerto Rico: Spleen eosinophils. (C) Flow cytometric analysis of spleen samples from mice infected with *Schistosoma mansoni* strain from Belo Horizonte, Liberia and Puerto Rico: Spleen dendritic cells. (D) Flow cytometric analysis of spleen samples from mice infected with *Schistosoma mansoni* strain from Belo Horizonte, Liberia and Puerto Rico: Spleen T-helper cells subtype 1. (E) Flow cytometric analysis of spleen samples from mice infected with *Schistosoma mansoni* strain from Belo Horizonte, Liberia and Puerto Rico: Spleen natural killer T-cells. (F) Flow cytometric analysis of spleen samples from mice infected with *Schistosoma mansoni* strain from Belo Horizonte, Liberia and Puerto Rico: Spleen T-helper cells. (G) Flow cytometric analysis of spleen samples from mice infected with *Schistosoma mansoni* strain from Belo Horizonte, Liberia and Puerto Rico: Spleen natural killer cells. (H) Flow cytometric analysis of spleen samples from mice infected with *Schistosoma mansoni* strain from Belo Horizonte, Liberia and Puerto Rico: Spleen T-helper cells subtype 17. (I) Flow cytometric analysis of spleen samples from mice infected with *Schistosoma mansoni* strain from Belo Horizonte, Liberia and Puerto Rico: Spleen regulatory T cells.
(XLSX)
